# Disruption of contactin 4 in three subjects with autism spectrum disorder

**DOI:** 10.1136/jmg.2008.057505

**Published:** 2008-03-18

**Authors:** J Roohi, C Montagna, D H Tegay, L E Palmer, C DeVincent, J C Pomeroy, S L Christian, N Nowak, E Hatchwell

**Affiliations:** 1 Department of Genetics, Stony Brook University, Stony Brook, New York, USA; 2 Department of Pathology and Molecular Genetics, Albert Einstein College of Medicine, Bronx, New York, USA; 3 Department of Pediatrics, Stony Brook University Medical Center, Stony Brook, New York, USA; 4 Department of Medicine & Medical Genetics, New York College of Osteopathic Medicine, Old Westbury, New York, USA; 5 Department of Microbiology, Stony Brook University, Stony Brook, New York, USA; 6 Department of Human Genetics, The University of Chicago, Chicago, Illinois, USA; 7 Department of Cancer Prevention and Population Sciences, RPCI and New York State Center of Excellence in Bioinformatics and Life Sciences, University at Buffalo, New York, USA; 8 Department of Pathology, Stony Brook University, Stony Brook, New York, USA

## Abstract

**Background:**

Autism spectrum disorder (ASD) is a developmental disorder of the central nervous system of largely unknown aetiology. The prevalence of the syndrome underscores the need for biological markers and a clearer understanding of pathogenesis. For these reasons, a genetic study of idiopathic ASD was undertaken.

**Methods and results:**

Array based comparative genomic hybridisation identified a paternally inherited chromosome 3 copy number variation (CNV) in three subjects: a deletion in two siblings and a duplication in a third, unrelated individual. These variations were fluorescence in situ hybridisation (FISH) validated and the end points further delineated using a custom fine tiling oligonucleotide array. Polymerase chain reaction (PCR) products unique to the rearrangements were amplified and sequence analysis revealed the variations to have resulted from Alu Y mediated unequal recombinations interrupting contactin 4 (*CNTN4*).

**Conclusion:**

*CNTN4* plays an essential role in the formation, maintenance, and plasticity of neuronal networks. Disruption of this gene is known to cause developmental delay and mental retardation. This report suggests that mutations affecting *CNTN4* function may be relevant to ASD pathogenesis.

Autism spectrum disorder (ASD) (Online Mendelian Inheritance in Man (OMIM) 209850) is a severe developmental disorder of the central nervous system characterised by impairments in three behavioural areas: (1) social interaction; (2) verbal and non-verbal communication; and (3) range of interests, activities and patterns of behaviour.^1^ The disorder is divided into five DSM-IV (*Diagnostic and statistical manual of mental disorders*, 4th ed) subtypes: autistic disorder, Asperger disorder, disintegrative disorder, pervasive developmental disorder not otherwise specified (PDD-NOS), and Rett disorder.^1^ With the exception of Rett disorder, reliable biological markers do not exist for diagnosis or classification.^2^ Given the prevalence of ASD (as high as 1 in 150 American children), a clearer understanding of aetiology is necessary both for diagnostic and therapeutic purposes.^3^

Pathogenesis of ASD has been argued to be both environmental and biological.^4^ Of the different biological causes associated with the disorder, genetic factors are the most important. Family studies demonstrate that the recurrence risk for an affected proband’s sibling is between 2–10%.^5^ This is 3–15 times the population risk for ASD.^3^ Same sex twin studies report an average concordance rate of 60–90% between monozygotic twins, compared with 0–10% in dizygotic twins.^6^ These twin findings support genetic inheritance as a major causative agent in ASD, but also suggest that epigenetic factors and exposure to environmental agents may contribute to the variable expression of autism traits, acting as modifiers in genetically susceptible individuals. The prevalence of communication disorders, social phobias and obsessive–compulsive disorder in non-autistic family members of autistic patients also implies that the expression of autism related traits is variable.^7^

This report describes copy number variations (CNVs) disrupting the same gene, contactin 4 (*CNTN4*), in three individuals with ASD. In each instance, the mutation resulted from an Alu Y mediated unequal recombination event. Alu elements have inserted into about one million sites of the human genome, accounting for about 10% of total genomic DNA.^8^ They are hotspots for recombination with Alu mediated rearrangement, resulting in approximately 0.3% of all genetic disease.^9^ The youngest subfamilies, including Y, Yc1, Yc2, Ya5, Ya5a2, Ya8, Yb8 and Yb9, have the greatest homology, and therefore are most likely to recombine.^9^

## METHODS

### Participants

Participants were recruited from the Cody Center for Autism and Developmental Disabilities clinic for an institutional review board approved genetics study. Initial evaluations for ASD had been performed by a multidisciplinary diagnostic team, supervised by an experienced developmental disabilities specialist (JCP). Evaluations included review of extensive behavioural, developmental and demographic information completed by caregivers and teachers, and a semi-structured parent interview. If, on further review for entry to this study, information regarding diagnosis was felt to be equivocal or incomplete, the Autism Diagnostic Observation Schedule (ADOS) and/or the Autism Diagnostic Interview-Revised were administered. A total of 92 subjects with ASD as well as both of their biological parents were enrolled and completed the study. Participants in the study came from 81 different families.

Individuals fulfilling the criteria for ASD underwent a physical examination at the General Clinical Research Center (Stony Brook University Medical Center (SBUMC)) conducted by a medical geneticist (DHT) and a blood sample was obtained from each subject for DNA isolation, conventional chromosome analysis (peripheral blood karyotype), fragile X testing, and, if indicated, Rett MECP2 testing. A blood sample was also obtained from the subject’s parents for DNA isolation. DNA isolation, peripheral blood karyotyping, and fragile X testing were performed by the Cytogenetics and Molecular Genetics Laboratories of the SBUMC using standard methods. Participants were excluded from the study if a Rett MECP2 or a fragile X mutation was discovered. DNA and medical records were coded to ensure confidentiality.

### Whole genome array comparative genomic hybridisation and fluorescent in situ hybridisation

**Figure 1:**
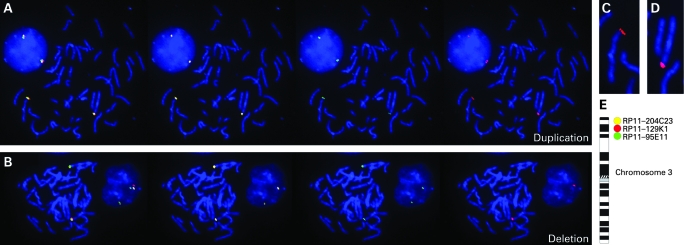
Fluorescence in situ hybridisation (FISH) validation of chr3p26.3 copy number variations (CNVs). (A) Validation of chr3p26.3 microduplication. FISH analysis confirmed the chr3p26.3 microduplication in subject 1 and his father (FISH images from subject 1 pictured). Panel order from left to right: composite image, left flanking clone (yellow), right flanking clone (green), clone within the CNV (red). (B) Validation of chr3p26.3 microdeletion. FISH analysis confirmed the chr3p26.3 microdeletion in 2B, 2C and their father (FISH images from subject 2C pictured). Panel order from left to right: composite image, left flanking clone (yellow), right flanking clone (green), clone within the CNV (red). (C) Normal chromosome 3. (D) Chromosome 3 with duplication. Comparison of red signal intensity in (C) versus (D) demonstrates a duplication. (E) Ideogram of clone locations. Three bacterial artificial chromosomes (BACs) were selected for each CNV: a left clone flanking the CNV pseudocoloured yellow (RP11-204C23), a right clone flanking the CNV pseudocoloured green (RP11-95E11), and clone within the CNV pseudocoloured red (RP11-129K1).

Genomic DNA from all subjects (and parents, in select cases) was hybridised onto tiling path bacterial artificial chromosome (BAC) arrays for copy number analysis as previously described.^10^ Image analysis was performed using the BlueFuse package (BlueGnome, Cambridge, UK). A subgrid loss corrected log2 ratio of the background subtracted test/control was calculated for each clone. Fluorescence in situ hybridisation (FISH) validation of genomic DNA CNVs was performed with Phi29 DNA polymerase amplified BAC clones as previously described.^11^ Three BAC clones were selected for each FISH experiment: (1) a left clone flanking the CNV (proximal to the centromere) was labelled with Biotin (Roche, Nutley, New Jersey, USA) detected with Streptavidin Cy5 (Jackson Immunoresearch Laboratories, West Grove, Pennsylvania, USA) and pseudocoloured in yellow; (2) a right clone flanking the CNV (distal to the centromere) was labelled with Spectrum Green dNTP (1.6 nmole, Vysis, Abbott Molecular Inc, Des Plaines, Illinois, USA) and pseudocoloured in green; and (3) clones within the change were labelled with Spectrum Orange dNTP (1.6 nmole, Vysis) and pseudocoloured in red (fig 1). In all, seven different clones from within the CNVs were tested (tables 1 and 2). Metaphase chromosomes from each subject were derived from normal B lymphocytes as previously described (www.riedlab.nci.nih.gov/protocols.asp). After hybridisation, slides were washed three times in 50% formamide and 2XSSC at 45°C and subsequently in 1XSSC at 45°C. Biotin labelled flanking clones were detected by incubating hybridised slides with a 1:200 dilution of Streptavidin Cy5 Ab. Slides were stained with DAPI and mounted with antifade (phenylene diamine).

**Table 1:** FISH validation of chr3p26.3 microdeletion

Region	BAC	Subject 2A	Subject 2B	Subject 2C	Mother	Father
Outside	RP11-204C23	Normal	Normal	Normal	Normal	Normal
Inside	RP11-129K1	Normal	Deleted	Deleted	Normal	Deleted
Inside	RP11-587N5	Normal	Deleted	Deleted	Normal	Deleted
Inside	RP11-35L22	Normal	Deleted	Deleted	Normal	Deleted
Inside	RP11-33J20	Normal	Deleted	Deleted	Normal	Deleted
Inside	RP11-129L14	Normal	Deleted	Deleted	Normal	Deleted
Inside	RP11-119L4	Normal	Deleted	Deleted	Normal	Deleted
Inside	RP11-916L15	Normal	Deleted	Deleted	Normal	Deleted
Outside	RP11-95E11	Normal	Normal	Normal	Normal	Normal

BAC, bacterial artificial chromosome; CNV, copy number variation; FISH, fluorescence in situ hybridisation.

FISH was performed on subjects 2A, 2B, 2C and their parents with 2 BACs flanking the CNV (RP11-204C23 and RP11-95E11) and a third BAC within the CNV.

**Table 2:** FISH validation of chr3p26.3 microduplication

Region	BAC	Subject 1	Mother	Father
Outside	RP11-204C23	Normal	Normal	Normal
Inside	RP11-129K1	Duplicated	Normal	Duplicated
Inside	RP11-587N5	Duplicated	Normal	Duplicated
Inside	RP11-35L22	Duplicated	Normal	Duplicated
Inside	RP11-33J20	Duplicated	Normal	Duplicated
Inside	RP11-129L14	Duplicated	Normal	Duplicated
Inside	RP11-119L4	Duplicated	Normal	Duplicated
Inside	RP11-916L15	Duplicated	Normal	Duplicated
Outside	RP11-95E11	Normal	Normal	Normal

BAC, bacterial artificial chromosome; CNV, copy number variation; FISH, fluorescence in situ hybridisation.

FISH was performed on subject 1 and his parents with 2 BACs flanking the CNV (RP11-204C23 and RP11-95E11) and a third BAC within the CNV.

Slides were imaged with an Olympus BX61 microscope with an UPlanSApo 100X NA 1.4 objective, an Hg arc lamp for excitation, and narrow band filters for all fluorescent emission. FISH image acquisition was done with a COOL-1300 QS SpectraCube camera (Applied Spectral Imaging, Vista, California, USA) using the FISHView software (version 4.0; Applied Spectral Imaging). Images of chromosome metaphases and interphase cells were acquired for each patient; a minimum of 10 metaphases and 10 interphase cells were analysed for each slide.

### Oligonucleotide microarray analysis

**Figure 2:**
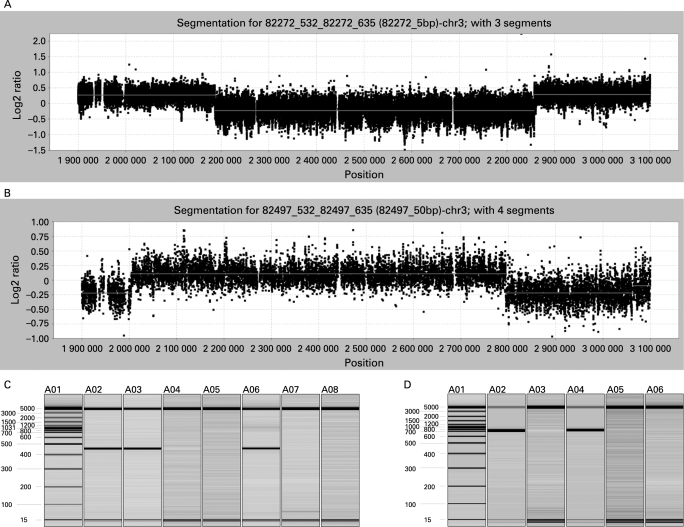
Fine Mapping of copy number variation (CNV) end points. (A) A NimbleGen fine tiling array further defines the deletion end points. A NimbleGen fine tiling array spanning positions 1 900 000–3 100 000 on chromosome 3 was used to map the deletion in greater detail. The log2 ratio (test/reference) deviates from 0 at positions ∼2 205 400–2 859 400, indicating the deletion breakpoints on chromosome 3. (B) A NimbleGen fine tiling array further defines the duplication end points. The same fine tiling array utilised in (A) was used to map the duplication in greater detail. The log2 ratio (test/reference) deviates from 0 at positions ∼2 003 900–2 795 000, indicating the duplication breakpoints on chromosome 3. (C) Polymerase chain reaction (PCR) across the deletion. PCR with primers spanning the deletion yielded a 454 bp product in subject 2C (A02), subject 2B (A03) and their father (A06). No product was present in subject 2A (A04), their mother (A05), a normal control (A07) or water (A08). See also fig 3A. (D) PCR across the duplication. PCR with primers between the duplicated regions on chr3 yielded a 520 bp product in subject 1 (A02) and his father (A04). No product was amplified from his mother (A03), a normal control (A05) or water(A06). See also fig 3B.

A custom 385 000 oligonucleotide NimbleGen fine tiling array spanning positions 1 900 000–3 100 000 on chromosome 3 was designed to map the variations in finer detail (fig 2A,B). Probes were selected from repeat masked sequence at an average spacing of 1.5 bp using previously described criteria.^12^ Hybridisations were performed at the NimbleGen Service Laboratory as previously described.^12^ Data were extracted from scanned images using NimbleScan version 2.3 extraction software. Extracted data were processed with SegMNT. Rearrangement breakpoints were determined by automated segmentation analysis of data sets after normalisation of signal intensities. The test versus reference log2 ratios were averaged at window sizes corresponding to 1× and 10× the median probe spacing.

### Polymerase chain reaction

**Figure 3:**
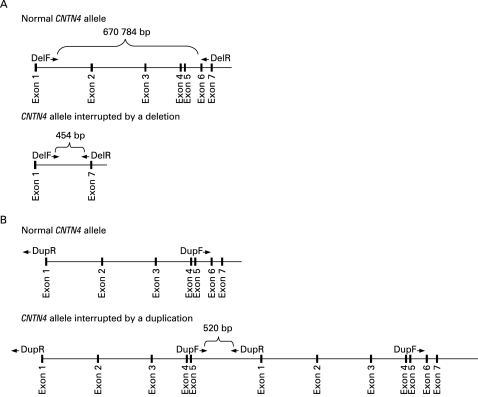
Diagram of polymerase chain reaction (PCR) primer design. (A) Amplification of chr3p26.3 deletion specific product. Primers DelF and DelR were designed to amplify a 454 bp product specific to the observed deletion. In normal genomic DNA, there primers are 670 784 bp apart and do not generate a product. (B) Amplification of chr3p26.3 duplication specific product. Primer DupF and Duper were designed to amplify 520 bp product specific to the observed tandem duplication. In normal genomic DNA, these primers are 790 018 bp apart and face opposite directions.

PCR primers (DelF-TGAGTTCACTACATGATGAGAGATAA and DelR-TCCAGTAGTCTCGCTTAAAAATTG) were designed from the deletion end points identified by oligonucleotide microarray analysis (fig 3A). Primers (DupF- GGCCAGCATATTTCTCCAAA and DupR-AAATCTGGCC GAAGTTCTGA) were designed from the duplication end points identified by oligonucleotide microarray analysis (fig 3B). PCR was performed on sample DNAs and on a control DNA sample known to be absent for the mutations. All PCR reactions were performed in a 50 μl mixture containing 50 ng DNA, 0.2 mM dNTP, 1 μM of each primer, 1.25 UHotMaster Taq polymerase, and 1XHotMaster buffer (Fisher Scientific, Pittsburgh, Pennsylvania, USA). Cycling was: 95°C for 5 min, followed by 30 cycles of 95°C for 30 s, 60°C for 30 s, and 68°C for 30 s, followed by a final extension at 68°C for 10 min. PCR reactions were analysed with an HDA-GT12 Genetic Analyzer (eGene Inc, Qiagen, Valencia, California, USA); products only amplified from individuals with the specific copy number change were analysed (fig 2C,D). PCR products were sequenced to determine the exact end points of the mutation.

### Alu analysis

**Figure 4:**
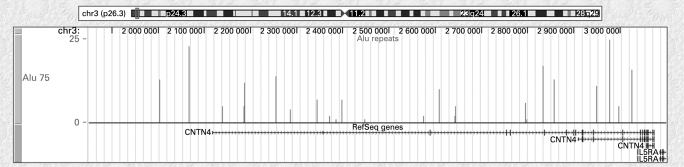
Histogram of Alu sequences near CNTN4. Alu sequences containing 100% identity of 75 bases or more to other Alu sequences within 5 Mb were plotted on the UCSC Genome Browser. The position of each Alu element found to match at least one other Alu, using this criterion, is denoted by a vertical bar. The height of each bar represents the number of “hits”.

Coordinates of Alu sequences within the human genome were extracted from the RepeatMasker track of the UCSC Genome Browser (March 2006 release).^13^ The coordinates were used to extract the DNA sequences from FASTA files downloaded from the UCSC genome database. Alu sequences were pairwise aligned using FASTA.^14^ For each Alu, the number of Alu elements within 5 Mb that contained at least one stretch of 75 bases at 100% identity was counted. These data were uploaded as a custom track onto the UCSC Genome Browser (fig 4).

## RESULTS

### Clinical reports

Subject 1 was evaluated for this study at the age of 7 years and 9 months with ASD. He was born full term following an uncomplicated prenatal course to non-consanguineous Caucasian parents. Family history was not significant. Parental concern first arose at 2 years of age due to speech delay. His first single words were noted between 18–24 months of age and phrase speech was absent until 3 years of age. He underwent brain magnetic resonance imaging (MRI) and electroencephalography (EEG) at 3 years of age which were reportedly normal. Seizures, regression, self injurious behaviours, auditory and visual deficits were absent. Initial evaluation for ASD was performed at age 2 (1996). At a follow-up examination at age 6, an ADOS was administered and subject 1 met cut-off for ASD with the following scores: communication  =  3; social  =  6; communication + social  =  9. On physical examination for this study, the subject’s height, weight and head circumference were at the 95th, >95th and 50th centile, respectively. Dysmorphic features included overfolded and low set ears, synophrys, mild hirsutism, a broad chest and narrow hands with a flat hypothenar eminence.

Subjects 2A, 2B and 2C are three non-twin full siblings who were evaluated, respectively, at the ages of 19 years 3 months, 15 years 11 months, and 10 years 11 months with ASD, again for this study. All were born full term following uncomplicated prenatal courses to the same non-consanguineous, reportedly non-autistic, Caucasian parents. The family history was significant for three unaffected paternal half siblings.

Subject 2A was a normally developing male until 18 months of age, with multiple single words and the ability to recite the alphabet (by parental report) before losing all speech and developing repetitive self stimulating behaviours. He underwent a brain MRI, head computed tomography (CT) and EEG at 4 years of age which were reportedly normal. Seizures, self injurious behaviours, auditory and visual deficits were absent. Initial evaluation for ASD was performed at age 3 years (1987). Subject 2A demonstrated a complete lack of speech, poor eye contact, and tried to remain distant in physical proximity from the clinician. He did not mimic any action or comply with any overt requests. Parents reported (on PDD checklist and in parent interview) an inability to socialise with peers or understand the emotions of others, lack of reaction to events (happy or sad), a fixation with ears and toes, lack of a sense of danger, flapped hands (particularly when excited) and extreme sensitivity to loud noises. At the time of evaluation for this study, the subject had regained some use of single words and short phrases. On physical examination (this study), his height, weight and head circumference were at the 25th, 5th and 75th centile, respectively. No significant dysmorphology was identified aside from a short philtrum, narrow hands and mild ligamentous laxity. He is the only sibling to have an early regressive course.

Subject 2B is a male who did not develop any speech until 4 years of age. He also developed seizures at 15 years of age. Regression, self injurious behaviours, auditory and visual deficits were absent. Initially this subject was evaluated for ASD at the age of 2 years (1990). On examination, the subject was restless and uncomfortable. There were no intelligible words but he did make sounds. He did not interact appropriately with toys and resisted eye contact and touching by interviewer. Parents reported immaturity for his age, separation problems, tantrums, resistance to demands, inability to recognise familiar objects, and lack of interactive play. On physical examination for this study, his height and weight were at the 25th and 50th centile, respectively. There were no significant dysmorphic features.

Subject 2C is a female noted to have speech delay at 2 years of age with minimal use of single words. Regression, seizures, self injurious behaviours, auditory and visual deficits were absent. An ADOS was performed at age 4 (1997). The subject met the cut-off for ASD: communication  =  4; social  =  6; communication + social  =  10. On physical examination for this study her height and weight were both above the 95th centile. Significant but not syndrome specific dysmorphic features included bilateral pre-auricular tags, mildly protruding ears, narrow hands, ligamentous laxity, obesity and a single ∼5 cm × 5 cm abdominal café-au-lait spot.

All four subjects were chromosomally normal and fragile X negative. Rett MECP2 testing was not indicated in any patient.

### Molecular analysis

Whole genome array comparative genomic hybridisation (aCGH) analysis of 92 subjects with ASD identified a CNV at 3p26.3 in three subjects. A deletion spanning ∼2 205 400–2 859 400 on chromosome 3 was detected in two siblings (subjects 2B and 2C) and a duplication spanning ∼2 003 900–2 795 000 on chromosome 3 was detected in a third unrelated individual (subject 1) (data not shown). Subsequent array analysis of parental DNA revealed that both variations were paternally inherited. FISH of metaphase spreads from both families confirmed the array findings (fig 1, tables 1 and 2). A custom oligonucleotide array spanning positions 1 900 000–3 100 000 on chromosome 3 was designed to map the changes in finer detail. Since the changes were inherited, the mutation end points where assumed identical within each family and only subject 2C and subject 1 were hybridised. The deletion was mapped to roughly chr3: 2 205 425–2 859 375 and the duplication to roughly chr3: 2 003 928–2 795 025 (fig 2A,B). This information was used to design primers unique to the CNVs. Two primers, normally 670 784 bp apart, amplified a 454 bp product specific for the chromosome 3 deletion (figs 2C and 3A). Sequence analysis revealed the deletion to have occurred between 2 ALU Y sequences at chr3: 2 186 724–2 187 030 and chr3: 2 857 104–2 857 410. Primers normally 790 018 bp apart were designed to map the duplication end points. Amplification yielded a 520 bp product specific to the CNV (fig 2D and 3B). Sequencing of the product revealed that the duplication resulted from of unequal recombination between an Alu Y at chr3: 2 004 085–2 004 406 and chr3: 2 794 618–2 794 952. The tandem duplication directly interrupts *CNTN4* (fig 3B).

Alu sequences from the region chr3: 1 850 000 to 3 100 000, which spans *CNTN4*, were analysed for homology to other nearby Alu elements. For each Alu in this region, a comparison was made with all other Alu elements mapping within 5 Mb (on either side). Those that contained at least 75 contiguous bases of 100% identity with another Alu were plotted (fig 4). Of the 356 Alus annotated in the UCSC genome database within this region, 25 were found to match one or more Alus within 5 Mb. All of these Alus are of the AluY (or one of its subtypes) class of Alus. This represents 43% of the AluY sequences in the region.

## DISCUSSION

The CNVs described in our subjects directly interrupt contactin 4 (*CNTN4*), an axon associated cell adhesion molecule (AxCAMs) highly expressed in the brain, particularly in the cerebellum, thalamus, amygdala, and cerebral cortex.^15^ Complex interactions between cell adhesion molecules (CAMs) are of critical importance during neurogenesis and the precise functioning of neural networks. AxCAMs are believed to play crucial roles in axonal elongation along specific pathways, fasciculation of specific axonal populations, and the formation, maintenance, and plasticity of some synaptic connections.^16^ The expression profile of *CNTN4* in human tissues indicates that the protein may have an important role in both the early growth of developing axons and in the maintenance of the adult nervous system. 16 In 2004, Fernandez *et al* reported a de novo balanced translocation, disrupting *CNTN4*, in a patient with the cardinal features of 3p deletion syndrome, including developmental delay and typical dysmorphic features.^15^ Other groups have also suggested that loss of a single functional copy of *CNTN4* contributes to the developmental delay characteristic of 3p deletion syndrome.^17^ ^18^

The syndrome is clinically recognised by a combination of features including growth and mental retardation, microcephaly, hypertonia, digital anomalies and dysmorphic facial features including a triangular shaped face, ptosis, hypertelorism, broad nasal root, long philtrum, down turned mouth, micrognathia and dysplastic ears.^15^ ^19^ ^20^ In this report, none of the subjects with CNVs interrupting *CNTN4* demonstrated the classical 3p deletion syndrome phenotype. Quite notably, growth retardation, microcephaly, digital anomalies, hypertonia and the characteristic facial gestalt were absent and only minor, non-specific dysmorphic features were identified. This is in contrast to previous reports of *CNTN4* deletion or interruption by translocation where aspects of the 3p deletion syndrome phenotype were described.^15^ As previously reported deletions involving *CNTN4* encompassed neighbouring genes, and as translocations may similarly result in position effects on neighbouring genes, it is intriguing to observe that the intragenic *CNTN4* CNVs identified in this report have been ascertained through the presence of a relatively isolated neurocognitive phenotype.^15^ Interestingly, mutations of contactin associated protein-like 2 (*CNTNAP2*) have also been linked to ASD and/or features of the syndrome, including seizures, language regression, and mental retardation.^21^^–^24 Therefore, given CNTN4’s vital role in both the development and maintenance of the nervous system, its implication in 3p deletion syndrome, and the correlation of associated proteins with autism, we believe mutations affecting the protein’s function may contribute to ASD pathogenesis.

In our subjects, the CNVs interrupting *CNTN4* were all inherited from fathers without a history of ASD; one consideration is that these CNVs are polymorphic and not pathologic. There have been a few rare reports of CNVs affecting *CNTN4* in normal individuals (Database of Genomic Variants, http://projects.tcag.ca/variation/).^25^ However, most of the described variations were detected with the Affymetrix 500K EA SNP Mapping Arrays, and, for the most part, have not been validated. It may be difficult to equate data from different platforms when it comes to copy number. In addition, the normal variation reported in the Database of Genomic Variants needs to be interpreted with care. The database lists several CNVs that would be expected to cause well known syndromes, including velo-cardio-facial syndrome, 22q13 deletion syndrome, and Sotos syndrome. It is also noteworthy that an ongoing study, in our own group, utilising the same BAC array platform and analysis methods applied with this cohort, failed to identify CNVs involving *CNTN4* in 560 National Institutes of Mental Health (NIMH) unrelated normal controls. Studies of other disorders (unrelated to ASD), also in our own group, have also failed to detect CNVs in this gene in 252 individuals (data not shown). If mutations of *CNTN4* are incompletely penetrant, disruption of the gene, although rare, may not result in ASD in all detected cases. The significance of an incompletely penetrant mutation is perhaps best examined between families instead of individuals. This study recruited 92 subjects from 81 different families. A CNV disrupting *CNTN4* is present in two families (∼2.5%). However, as mentioned above, BAC microarray analysis did not detect a variation affecting *CNTN4* in normal controls (data not shown). Assuming each normal individual is representative of a different family, statistical analysis of these findings with a Fisher Exact Test (http://www.physics.csbsju.edu/stats/fisher.form.html) indicates loss of one functional copy of *CNTN4* is a significant contributing factor to the development of ASD (p = 0.016). Although these results need further confirmation in larger cohorts, they strongly suggest that mutations affecting *CNTN4* function can cause ASD, despite their detection in a small number of reportedly normal individuals. Notably, incomplete penetrance has been described in other ASD associated mutations, including a chromosome 16p11.2 CNV that reportedly accounts for approximately 1% of all cases of the syndrome.^26^ The CNV was a de novo event in most subjects, but additionally was inherited in some individuals from an unaffected parent. It was also found to occur rarely in normal controls as well.

It is noteworthy that studies of large scale CNV in the human genome, to date, have not examined their cohort’s family history. Given the prevalence of ASD (1 in 150 American children), it is possible that “normal” individuals with CNVs affecting *CNTN4* come from families with the disorder. Had the two fathers described in our study been part of a normal cohort, it is unlikely that the presence of ASD in their children would have been noted. Consider the case of thrombocytopenia-absent radius (TAR) syndrome. A recent study of the disease identified a 200 kb deletion on chromosome 1 in all 30 TAR patients examined.^27^ In a majority of cases, the deletion was inherited from an unaffected parent but the variation was completely absent from a group of 700 normal individuals. This suggests that the deletion contributes to TAR syndrome but the phenotype only develops in the presence of an as-yet-unknown modifier. Disruption of *CNTN4* may affect the development of ASD in a similar manner. Imprinting, environmental interactions, or other factors may determine how mutations in *CNTN4* cause ASD.

**Key points**

- A genetic study of autism spectrum disorder (ASD) identified paternally inherited copy number variations (CNVs) of chr3p26 in three individuals with the disorder.
- The CNVs affected one gene directly, contactin 4 (*CNTN4*). *CNTN4* participates in neurogenesis and functioning of neural networks. Disruption of this gene is known to cause developmental delay and mental retardation.
- Molecular characterisation of the CNVs revealed that they resulted from Alu Y mediated unequal recombination.

It is worth mentioning that subject 2A has ASD but does not carry the chromosome 3 CNV. However, he is the only child in his family to have an early regressive course, suggesting perhaps that his disease is somehow different from his siblings. Also of interest is the fact that this phenomenon has been described in ASD before. The Autism Research Consortium observed a 22q11.2 duplication in two multiplex families. In one, it was inherited from a parent, while in the other it was de novo.^28^ In both families, only one child diagnosed with ASD carried the duplication. Alarcon *et al* describe a large intronic deletion in a multiplex family inherited in one autistic sibling but not the other.^22^ Given the frequency of ASD in the general population, it is possible that, on occasion, individuals from the same sibship may have the syndrome for different reasons.

Both of the CNVs interrupting *CNTN4* resulted from Alu Y mediated unequal recombination. Rearrangements involving Alus, in general, are most likely to occur between repeat elements on the same chromosome located within 5 Mb of each other with high sequence identity.^29^ The Alu Y mediated rearrangements reported here fit these criteria, as do 43% of the Alu Y elements around *CNTN4*. The high degree of Alu Y homology in this region perhaps predisposes it to Alu Y mediated unequal recombination. However, although Alu density may contribute to these recombination events, other factors likely influence the rearrangements. Analysis of other Alu-rich genes has found there is not a direct correlation between Alu density and recombination rate.^30^ Some other factors likely contribute to the recombination events observed in our subjects and the high Alu Y density in the region likely aids in the process.^30^

Our work implicates *CNTN4* as a candidate gene in ASD. Ongoing efforts are underway to sequence the gene in large numbers of subjects with ASD and normal controls to identify subtle mutations that might be involved in pathogenesis.
